# Interoceptive Focus Shapes the Experience of Time

**DOI:** 10.1371/journal.pone.0086934

**Published:** 2014-01-29

**Authors:** Olga Pollatos, Jochen Laubrock, Marc Wittmann

**Affiliations:** 1 Health Psychology, Institute of Psychology, University of Ulm, Ulm, Germany; 2 Department of Psychology, University of Potsdam, Potsdam, Germany; 3 Department of Empirical and Analytical Psychophysics, Institute for Frontier Areas in Psychology and Mental Health, Freiburg, Germany; University G. d'Annunzio, Italy

## Abstract

The perception of time is a fundamental part of human experience. Recent research suggests that the experience of time emerges from emotional and interoceptive (bodily) states as processed in the insular cortex. Whether there is an interaction between the conscious awareness of interoceptive states and time distortions induced by emotions has rarely been investigated so far. We aimed to address this question by the use of a retrospective time estimation task comparing two groups of participants. One group had a focus on interoceptive states and one had a focus on exteroceptive information while watching film clips depicting fear, amusement and neutral content. Main results were that attention to interoceptive processes significantly affected subjective time experience. Fear was accompanied with subjective time dilation that was more pronounced in the group with interoceptive focus, while amusement led to a quicker passage of time which was also increased by interoceptive focus. We conclude that retrospective temporal distortions are directly influenced by attention to bodily responses. These effects might crucially interact with arousal levels. Sympathetic nervous system activation affecting memory build-up might be the decisive factor influencing retrospective time judgments. Our data substantially extend former research findings underscoring the relevance of interoception for the effects of emotional states on subjective time experience.

## Introduction

During the last decade, the concept of embodiment has risen to become a key paradigm of interdisciplinary approaches from the areas of philosophy, psychology, psychiatry and neuroscience. The concept of embodiment refers to the intrinsic role the body has in shaping the mind and is based on a remarkable convergence of phenomenology, cognitive science and dynamical systems theory. Embodiment implies the close connection between brain processes, whole bodily functions and aspects of the mind such as consciousness, emotion, cognition, and self-awareness [1].

The idea that we feel emotions because we perceive our bodily reactions is a core characteristic of many so-called peripheral theories of emotion. William James [2] was one of the first to present a psychological theory linking viscero-afferent feedback to emotional experience. He suggested that a situation initiates specific visceral, vascular or somatic activities, such as changes of blood pressure and heart rate which are experienced and interpreted as emotions. Refinements of this model include Damasio's notion of somatic markers, which represent involuntary changes in bodily states signaling stimulus significance to guide both emotional and cognitive behavior (e.g. decision-making) [3], [4]. Several studies have demonstrated that a more pronounced awareness for interoceptive signals is associated with more intense feelings and higher activation of underlying brain structures during emotional stimulation [5]–[7]. Interoception and awareness for interoceptive signals may thus modulate the relationship between bodily responses, affective and cognitive processes which is in accordance with the pivotal role in introduced concepts of embodied cognition.

Time perception is fundamental for human experience; it is essential for everyday behaviour and for understanding complex behaviour [8]. Related to the embodiment concept, a direct link between the perception of time and body processes was proposed by Craig [9], who claims that our experience of time relates to emotional and visceral processes because they share a common underlying neural system, the insular cortex and the interoceptive system. There is evidence that primates represent homeostatic afferent activity that reflects the physiological condition of the body in “interoceptive centers” in the brain, especially the insular as well as medial prefrontal and orbitofrontal cortices [10]. Interoception is defined as the perception of signals arising from within the body [7], [11].

In this context, Craig [9], [12] proposes that body signals as accumulated in the insular cortex underlie our perception of duration. According to this conceptualization, neural substrates responsible for conscious awareness across time are based on the neural representation of the physiological condition of the body [9], suggesting a close connection between interoceptive processes and time perception [9], [12], [13]. Our experience of time thereafter emerges from emotional and visceral states processed in the insular cortex. However, there is only sparse empirical evidence supporting this assumption. Among these findings, two recent functional MRI studies have shown climbing activation in the insular cortex to correspond to stimulus length in a duration reproduction task, this finding being discussed as indicative of the insular cortex role in the perception of time [14], [15]. Moreover, one psychophysiological study by Meissner and Wittmann [16] demonstrated that individuals' duration reproduction accuracy correlated positively both with cardiac parameters (the slope of cardiac slowing during the encoding of temporal intervals) and with individuals' awareness of their own heart-beat. The results were interpreted as supporting the view that autonomic function and interoceptive processes underpin our perception of time intervals in the range of seconds.

It is important to note that emotions crucially interact with the experience of time: the accuracy and precision of time estimation in the seconds-to-minutes range is under the influence of cognitive and emotional factors, usually discussed in the framework of standard cognitive models which propose a pacemaker–accumulator that is embedded in a system of cognitive components [13], [17]. Within this model, emotions can be seen as associated with increased physiological arousal levels that lead to a higher pacemaker rate of an assumed internal clock (e.g. [18]). A higher pacemaker rate in turn leads to the accumulation of more temporal units during a given time span reflected by an overestimation of subjective duration and by the feeling of a slower passage of time [19]. This theoretical assumption is reflected by empirical data demonstrating that highly arousing, unpleasant static pictures or film clips typically elicit a relative overestimation of subjective duration (or “time dilation”) [20]–[23], whereas in an emotionally positive situation subjective time often contracts (see [9]).

In this context, Craig has formulated an asymmetric model of homeostatic emotion regulation, in which the left forebrain is associated predominantly with parasympathetic activity (related to positive affect, affiliative feelings and approach behavior), and the right forebrain is associated predominantly with sympathetic activity (related to negative affect, arousing feelings). In a sympathetically arousing situation the neural representational activity for global emotional moments would accumulate rapidly in the right anterior insula leading to subjective time dilation and the feeling that time passes slowly [9].

In situations in which the parasympathetic tone increases, the neural representational activity for global emotional moments in the left anterior insula would progress more slowly. Then, subjective time contracts and seems to pass relatively more quickly [9].

As summarized by e.g. Kreibig [24], different emotions are characterized by distinct autonomic nervous system patterns. Physiological responding to fear points to broad sympathetic activation, including cardiac acceleration, increased myocardial contractility, vasoconstriction, and increased electrodermal activity [24]. All positive emotions with the exception of joy are characterized by decreased sympathetic influence. The autonomic response pattern of amusement – in experimental settings typically induced by film clips – is characterized by increased cardiac vagal control, respiratory, and electrodermal activity, together with sympathetic deactivation. Heart rate variability is often increased, while heart rate results are less univocal with heart rate found to be decelerated, unchanged or even accelerated [24]. Focusing on the two emotional qualities of fear and amusement, it can be followed that fear should be associated with subjective time dilation, while amusement should lead to subjective time contraction. Furthermore, it can be hypothesized that when one pays attention more strongly to interoceptive signals a more intensive perception of bodily changes is induced which might emphasize the proposed effect of emotional states on subjective time experience. This would be in accordance with data suggesting that participants who are more aware of their bodily signals experience emotions more intensely [6], [25] and show a more pronounced central processing (as assessed by greater P300 amplitudes) of emotional stimuli [6]. Moreover, individuals who were more accurate in counting their heartbeats in the heartbeat perception task were also more accurate in time estimation [16].

Taking these lines of evidence into account the main aim of this study was to examine whether interoceptive processes, respectively attending to interoceptive processes, would affect time perception in an emotional stimulation paradigm. We decided to use a retrospective time estimation paradigm and induce the emotional qualities of amusement and fear by the use of film clips with duration of 40 seconds. In a retrospective paradigm, a person is not aware that a duration judgment has to be made until requested. Although subjective time is often tested in prospective time estimation situations, i.e. an individual is aware that he or she has to estimate the duration of events, we chose a retrospective time estimation test. Only rarely and in very special circumstances does one estimate temporal intervals with multiple-seconds duration prospectively. Related to the employed task, we normally do not wait through different intervals with an explicit instruction to judge duration. Rather, events with longer duration in the multiple-second range are often judged in retrospect, i.e. when they have already passed and we state that event *a* seemed to last longer than event *b*.

It has been shown that remembered duration depends strongly on incidental memory for temporal information and is modulated by variables such as arousal or emotional content [26],[27]. Generally speaking, remembered duration depends upon changes in context, upon the memory for changes of formerly experienced events. Changes in emotional context during temporal intervals with complex event structure will elicit changes in bodily and emotional reactions that might influence retrospective time. To allow participants to fully concentrate on bodily processes, we decided to use this experimental procedure in order to maximize assumed interaction effects between interoceptive signal processing and emotion induction on the experience of time. We hypothesized that both, time dilation and time contraction, are more pronounced in the group with interoceptive focus as compared to an exteroceptive focus.

## Materials and Methods

### Ethic statement

Experiments were conducted in accordance with the Declaration of Helsinki with the approval of the local ethics committees (ethic committee of the Department of Psychology at the University of Potsdam). In accordance with the local ethic committee all participants provided their written informed consent.

### Participants

254 participants were screened for health status using a medical history questionnaire. They were only included if they did not have a history of any axis 1 disorders, in particular anxiety disorders or depression according to the Diagnostic and Statistical Manual of Mental Disorders. Drug use (with the exception of contraceptives) was not allowed. Participants were either recruited and assessed at the University of Potsdam (N = 121; April to June 2012) or at the University of Ulm (N = 133; October to December 2012). All participants gave their written informed consent and received a course credit for their participation. 211 participants (55 male; mean age 21.4, SD 3.1) were included in the main experiment.

### Stimuli

Three film clips (neutral content: scene from a documentary description of a city in Germany; fear content: horrifying scene from the movie “Blair witch project”; amusement content: funny scene from the movie “Ice age part III”; duration 40 seconds each) served as emotional stimuli.

### Procedure

All experiments took place between 10 and 12 a.m. Upon arrival in the seminary rooms, participants filled out the anamnestic questionnaire and received randomly one of two instructions resulting in about 50% of participants assigned to each condition. These two conditions were (a) “interoceptive focus”: participants were instructed to carefully pay attention to their body and bodily reactions that might occur during watching the film clips (N = 105); (b) “exteroceptive focus”: they were instructed to carefully attend to details in the film clips as they would be asked to answer some questions afterwards (N = 106). Participants were instructed not to look away or distract themselves.

Data were assessed in four different experimental sessions (corresponding to the four courses the students were attending) with 43 to 68 participants in each of these sessions. In each session, the order of the film clips presented was randomized. After the first film clip, participants had to answer two questions: In the condition “interoceptive focus” they were asked to provide valence and arousal ratings on a 9-point scale using a non-verbal pictorial self-report, the Self-Assessment Manikin (SAM) [28]. Participants were asked to rate how pleasant vs. unpleasant and how aroused vs. calm they felt while watching the movie clips with scores ranging from 1 (very unpleasant or low arousing) to 9 (very pleasant or high arousing). In the condition “exteroceptive focus” they were asked two questions on certain aspects of the film clips (e.g. How many people were involved? Was a man or a woman the main actor? Which animals were depicted?). The rating period lasted 10 seconds before the next movie clip was presented, again followed by a rating period, and then the last film clip was presented. At the end of the third rating period all participants were asked to estimate the total length (in seconds) of each movie clip presented before. The whole experiment lasted about twenty minutes.

### Data analyses

Time length (in seconds) as well as total difference in estimated duration between the neutral film clip and the two emotional ones were examined using ANCOVAs with two levels of Condition (interoceptive/exteroceptive focus) and three (neutral, amusement, fear) respectively two (neutral minus amusement, neutral minus fear) levels of Emotional Content, Age was used as covariate.

Subjective valence and arousal were examined in the group with “interoceptive focus” only using ANCOVAs with three levels of Emotional Content (neutral, fear, amusement), Age was used as covariate.

Pearson partial correlation analyses (effects of age were controlled) were calculated between subjective valence and arousal and corresponding change in time experience (neutral minus amusement, fear minus neutral). In a last step, two hierarchical regression analyses with perceived time distortion as criterion (during positive and negative film presentation) and a. valence, b. arousal, c. age as predictors were calculated. In the results section, uncorrected F-values are reported together with the Greenhouse-Geisser epsilon values and Bonferroni corrected probability levels.

## Results

### Subjective experience of time

#### Absolute time length

**Figure 1:**
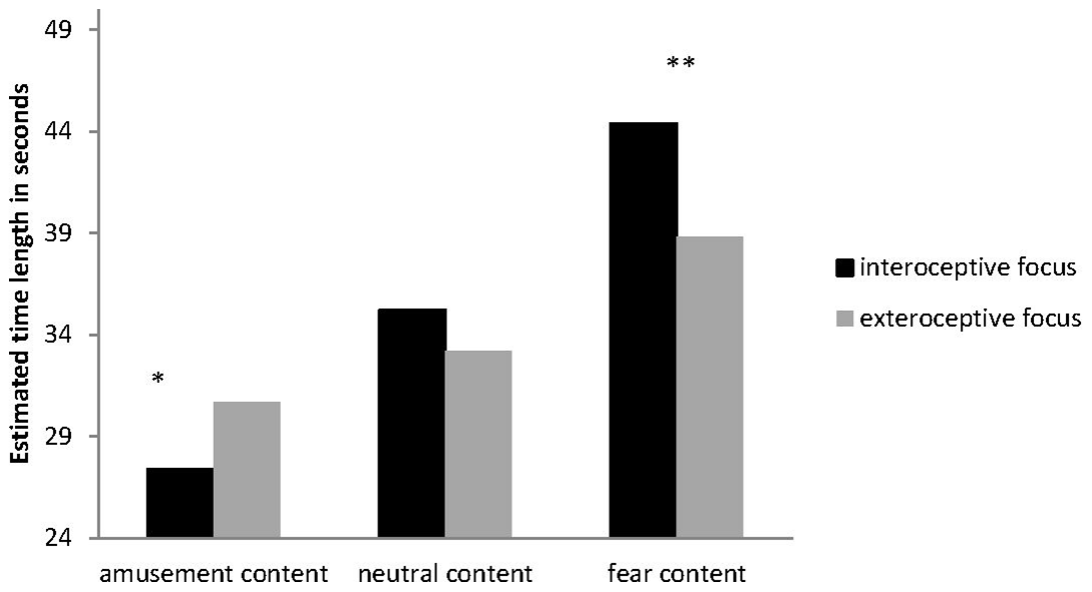
Estimated time lengths (in seconds) contrasting interoceptive and exteroceptive focus (*: p<.05; **: p<.01; bars represent standard errors).

Estimated time lengths (in seconds) contrasting both conditions are depicted in Figure 1 and summarized in Table 1.

**Table 1:** Descriptive values (mean, standard error/SE) and corresponding statistical test scores focusing on group differences (*: p<.05; **: p<.01; ***: p<.001).

	amusement condition (mean ± SE)	neutral condition (mean ± SE)	fear condition (mean ± SE)
	*Interoceptive focus group*		
Absolute time (in s) estimation	27.4 (1.1)	35.2 (1.1)	44.4 (1.5)
Relative difference scores time estimation	7.8 (1.0)	–	−9.2 (1.2)
Subjective Arousal	5.9 (0.1)	4.7 (0.1)	6.5 (0.2)
Subjective Valence	7.6 (0.1)	5.3 (0.1)	3.7 (0.1)
	*Exteroceptive focus group*		
Absolute time (in s) estimation	30.7 (1.1)	33.2 (1.1)	38.8 (1.5)
Relative difference scores time estimation	2.4 (1.0)	–	−5.6 (1.1)
	*ANCOVA focussing on group differences*		
Absolute time (in s) estimation	F(1,208) = 5.84	F (1, 208) = 1.65	F(1, 208) = 7.10
Significance	*	p = .20	**
Relative difference scores time estimation	F (1, 208) = 13.66	–	F(1, 208) = 4.98
Significance	***		*

*Note*: Subjective valence and arousal only assessed in the interoceptive focus group. Main effects of emotional content or interaction effects between group and emotional content are described in the text and not reported in this table.

The ANCOVA revealed a significant main effect of Emotional Content (F (2, 416)  = 3.55; p<.05; η^2^ = .02, ε = .65). Subjective duration was significantly longest for fear content (mean 41.5 s), followed by neutral content (mean 34.2 s). Amusement content (mean 29.1 s) had the shortest subjective duration (all p<.001). Additionally, we obtained a significant interaction, Emotional Content × Group (F (2, 416)  = 13.96; p<.001; η^2^ = .06; ε = 1.00), indicating that exteroceptive vs. interoceptive focus had a differential effect on subjective time experience depending upon the emotional condition. Age (F (1, 208) = 1.39, p = .24) or the interaction Age × Emotional Content (F (2, 416)  = 1.54, p = .22) were not significant.

Post hoc ANCOVAs for each emotional content with Age as covariate demonstrated that fear content had a significantly longer (F(1, 208)  = 7.10, p<.01) subjective duration in the condition “interoceptive focus” as compared to the “exteroceptive focus” (see Table 1). Amusement content had a significantly shorter (F(1, 208)  = 5.84, p<.05) subjective duration in the “interoceptive focus” condition as compared to the “exteroceptive focus”. No significant difference between both groups occurred for neutral content (F(1, 208)  = 1.65, p = .20; compare Table 1).

#### Relative time length

**Figure 2:**
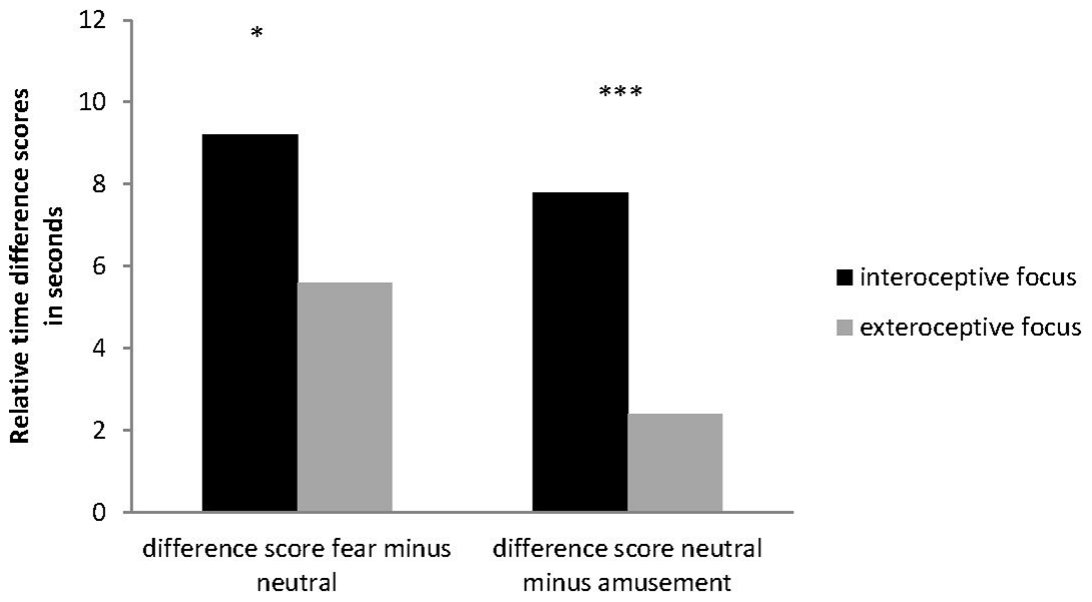
Relative time difference scores (in seconds) between a. fear minus neutral content, and b. neutral minus amusement (*: p<.05; ***: p<.001; bars represent standard errors).

To account for the fact that the subjective duration of the neutral film clip (mean 34 s) was also shorter than physical time (duration 40 s), we calculated difference scores (amusement content minus neutral/fear content minus neutral). Figure 2 depicts the estimated absolute time differences in seconds contrasting both groups. Table 1 summarizes all values.

The ANCOVA revealed a significant main effect of Emotional Content (F (1,208)  = 4.92; p<.05; η^2^ = .02; ε = .60) indicating positive difference scores for fear (subjective time dilation) and negative difference scores for amusement content. Age (F (1, 208) = 0.61, p = .43) or the interaction Age × Emotional Content (F (2, 416)  = 1.95, p = .17) were not significant. A significant interaction Emotional Content × Group (F (1,208)  = 19.80; p<.001; η^2^ = .09; ε = .99) was observed. Post hoc ANCOVAs for both difference scores separately showed that the relative time dilation for fear content (F(1, 208)  = 4.98, p<.05) and the relative time contraction for the amusement condition (F(1, 208)  = 13.66, p<.001) were more pronounced in the interoceptive focus as compared to the exteroceptive focus group (see Table 1).

### Feelings during emotional film presentation and time experience

Feelings during emotional film presentations were assessed with respect to subjective valence and subjective arousal in the interoceptive focus group only. All results are summarized in Table 1.

With respect to *valence ratings*, the statistical analysis revealed a significant effect for Emotional Content (F (2, 202)  = 3.90; p<.05; η^2^ = .04; ε = .68). Positive valence during pleasant film presentation (mean 7.7) was significantly higher than during neutral film presentation (mean 5.3) which was in turn significantly higher than during unpleasant film presentation (mean 3.7; p<.001). A significant interaction Age × Emotional Content (F (2, 202)  = 4.26; p<.05; η^2^ = .04; ε = .72) was observed: Post hoc correlational analyses showed that a higher age was associated with a smaller and therefore more unpleasant rating for neutral content only (r = −.24, p<.01), while no significant correlation occurred between age and unpleasant (r = .08, p = .29) as well as pleasant (r = .06, p = .47) content.

Concerning *subjective arousal*, a significant main effect of Emotional Content (F (2, 204)  = 4.89; p<.05; η^2^ = .05; ε = .75) occurred. Subjective arousal during neutral film presentation (mean 4.7) was significantly lower than during the amusement film presentation (mean 5.9, p<.001) which in turn was significantly lower than subjective arousal during the fear movie clip (mean 6.5; p<.01). A significant interaction Age × Emotional Content (F (2, 204)  = 3.48; p<.05; η^2^ = .03; ε = .59) was observed: Post hoc correlational analyses revealed a trend showing that higher age was associated with less arousal for unpleasant content (r = −.19, p = .053). No significant correlation occurred between age and neutral (r = .08, p = .42) as well as pleasant (r = .10, p = .31) content.

#### Correlations and regression analyses between subjective temporal distortion and subjective valence/arousal

Partial correlation coefficients were calculated between subjective valence and subjective arousal with the corresponding change in time experience (neutral minus amusement, fear minus neutral) for the participants with interoceptive focus. We controlled for influences of Age in all analyses. Main results are visualized in Table 2.

**Table 2:** Partial correlation coefficients between temporal distortion and subjective valence/arousal in the interoceptive focus group (*: p<.05, **: p<.01).

	Relative difference score neutral minus amusement	Relative difference score fear minus neutral
Subjective Arousal	r = .14	r = .31
Significance	p = .16	**
Subjective Valence	r = 0.23	r = 0.00
Significance	*	p = .99

*Note*: Influence of age was controlled.

We obtained a significant correlation between subjective arousal and subjective time dilation during the fear movie clip (r = .31, p<.01) indicating that a higher arousal was associated with larger time dilation (see Table 2). Additionally, a significant positive correlation coefficient of r = .23 (p<.05) between valence and the corresponding change in time experience (neutral minus amusement) was observed: A higher pleasantness rating was associated with a stronger contraction of subjective time. All other correlation coefficients were not significant (compare Table 2).

As subjective feelings measured by perceived arousal and valence are interconnected, we performed two hierarchical regression analyses (forward stepping) focusing on perceived time distortion as criterion (during positive and negative film presentation) and a. subjective valence, b. subjective arousal, c. age as predictors. *Subjective time dilation during fear* was explained by differences in perceived arousal only (T = 3.26, β = .31, F(1,102) = 10.65, p<.01, R = .31, R^2^ = .10), *subjective time contraction during amusement* was explained by differences in perceived valence (T = 2.16, β = .21, F(1,102) = 4.67, p<.05, R = .21, R^2^ = .04). All other predictors were not included.

## Discussion

In accordance with our hypotheses, attention to interoceptive processes significantly affected subjective time experience. A movie clip eliciting fear was accompanied with subjective time dilation that was more pronounced in the group with interoceptive focus. The amusement situation led to a quicker passage of time which was also modulated by interoceptive focus. Additionally, correlation and regression analyses indicated that higher arousal was associated with relative longer estimates of duration for fear content only. Concerning amusement, a higher pleasantness rating was associated with a stronger contraction of time passing. Our findings suggest that the more aware a person is of ongoing bodily processes, the more pronounced is the influence of emotional states on subjective time experience. These effects crucially interact with subjective arousal ratings for unpleasant content, while an increase in positive valence rather than in subjective arousal accounted for changes in time experience for pleasant content.

Our data provide new evidence for the relevance of interoceptive signal processing for the effect of emotional states on time experience. Both an unpleasant emotion like fear as well as a positive emotion like amusement affected retrospective time estimates, but these effects were stronger when participants were instructed to sense bodily changes while watching the film clips. Our results support the view that physiological states and emotions associated with changes in physiological states as hypothesized in peripheral theories of emotions influence subjective time estimates. While there is some empirical evidence for the link between interoceptive processes and time experience in prospective time paradigms [16], [21]–[23], this work extends previous investigations by demonstrating that such an association also exists for retrospective time estimation. Retrospective time estimates depend upon incidental memory for temporal information [26], that is, in previous studies interoceptive awareness was found to be associated with increased incidental memory load [29] and higher arousal levels for emotional content [5], [6], [30], [31]. It can be assumed that higher arousal and an associated higher degree of incidental memory during fear induction thus might have led to a stronger retrospective time dilation effect in the interoceptive focus group.

Referring to the observed stronger time contraction induced by amusement in the interoceptive focus group a different mechanism must be hypothesized. Craig assumes that if parasympathetic tone increases heart rate variability – such as typically induced by amusement (see [24]) – so called global emotional moments represented in both the left and right anterior insula progress slowly because of low sympathetic salience [9]. Amusement such as induced in this investigation typically increases cardiac vagal control together with sympathetic deactivation, while fear leads to a broad sympathetic activation, including cardiac acceleration, increased myocardial contractility, vasoconstriction, and increased electrodermal activity [24]. Previous research showed that autonomic reactivity was positively correlated with interoceptive awareness [29], [32]–[34], allowing us to hypothesize that an interoceptive focus while watching film clips with positive valence might be related to a more pronounced decrease of sympathetic salience. This assumption is supported by the fact that changes in valence (rather than in arousal) are related to subjective time contraction during the amusement condition. It has to be considered that the regression analysis showed that the amount of explained variance in time contraction by emotional valence is rather small, suggesting that additional variables may play a role. As we did not assess autonomic activity in this study we can only speculate about the detailed mechanisms underlying the observed interaction between interoceptive processes, emotional states and the subjective experience of time. Further research has to focus on these processes and try to disentangle the separate proportions of variance that might contribute to the observed effects.

Potential weaknesses of the current study refer on the one hand to that fact that we examined subjective valence and arousal only in the interoceptive group. This procedure was chosen to minimize introspection in the exteroceptive focus group and is therefore a design feature. It would have been interesting to see whether subjective arousal also plays a role for the time distortion effect in the exteroceptive focus group as our data suggest for the interoceptive focus group. A way to manage this problem in following studies might be to introduce several levels of interoceptive focus instead of creating an exteroceptive condition which is affected – to a certain extent – by memory and attention processes. It therefore cannot be ruled out that the exteroceptive condition was influenced by e.g. individual differences in memory capacity that might interact with the intended contrast between interoceptive and exteroceptive focus. On the other hand, we assessed time estimation using video clips instead of using other stimulus material such as tones or static pictures. This was mainly due to the fact that we wanted to use a retrospective time estimation task characterised by a lack of overt awareness for the passage of time and use an experimental design known to induce a strong emotional response. A third point that would be interesting to address in future studies is the question whether there is an effect or an interaction between age and subjective time distortion. We controlled for age in all our analyses and did not obtain any significant effect or interaction of age on time perception scores. Nevertheless, there was a significant effect of age on valence, and hereby on the estimated valence of neutral content, and also a trend towards a modulation of arousal. Therefore it might be the case that when including participants with a higher age, and possibly from a different social background, we might gain further insights.

A focus on bodily signals while viewing emotional content modulates retrospective duration. While most conceptual frameworks on the association between interoception and time experience so far have focused on prospective time perception, i.e. when participants are actually attending throughout the presented interval to time [8], [14], [16], we extend these findings by showing that also a retrospective time estimation task is crucially influenced by interoceptive processes. This is in accordance with former research on the interaction of emotion processing and interoception where in several studies individual differences in interoceptive awareness mediated the awareness and bodily response pattern to emotional stimuli even if these were processed incidentally [5], [29], [32]. The important role of interoceptive signals on mental processing is emphasized in several theories proposing the embodiment of emotional and cognitive processes [35]–[37]. In line with these theories interoception has become a main topic in recent attempts to explain mechanisms of time perception. Our data support this view and substantially extend former research underscoring the relevance of interoception for the interaction of emotional states on retrospective time experience. Moreover, we have demonstrated that subjective temporal distortions are directly influenced by attention to bodily responses. We conclude that bodily states and their meta-representation as somatic markers are an essential context variable for the effect of emotional states on subjective time distortion.
